# Exercise Intolerance and Oxygen Desaturation in Patients with Parkinson’s Disease: Triggers for Respiratory Rehabilitation?

**DOI:** 10.3390/ijerph182312298

**Published:** 2021-11-23

**Authors:** Michele Vitacca, Adriana Olivares, Laura Comini, Giuliana Vezzadini, Annamaria Langella, Alberto Luisa, Anna Petrolati, Gianluigi Frigo, Mara Paneroni

**Affiliations:** 1 Respiratory Rehabilitation of the Institute of Lumezzane, Istituti Clinici Scientifici Maugeri IRCCS, 25065 Lumezzane, Italy; mara.paneroni@icsmaugeri.it; 2 Scientific Direction of the Institute of Lumezzane, Istituti Clinici Scientifici Maugeri IRCCS, 25065 Lumezzane, Italy; adriana.olivares@icsmaugeri.it (A.O.); laura.comini@icsmaugeri.it (L.C.); 3 Neurorehabilitation of the Institute of Castel Goffredo, Istituti Clinici Scientifici Maugeri IRCCS, 46042 Castel Goffredo, Italy; giuliana.vezzadini@icsmaugeri.it (G.V.); annapetrolati75@gmail.com (A.P.); gianluigi.frigo@icsmaugeri.it (G.F.); 4 Neurorehabilitation of the Institute of Lumezzane, Istituti Clinici Scientifici Maugeri IRCCS, 25065 Lumezzane, Italy; annamaria.langella@icsmaugeri.it (A.L.); alberto.luisa@icsmaugeri.it (A.L.)

**Keywords:** Parkinson’s disease, respiratory function, exercise tolerance, oxygen saturation, rehabilitation

## Abstract

The role that oxygen desaturation plays in exercise tolerance and its rehabilitative implications in patients with Parkinson’s disease (PD) are unclear. We aimed to test exercise tolerance and oxygen saturation levels both during exercise and at night in PD patients to better define their rehabilitative needs. In clinically stable PD patients, undergoing inpatient rehabilitation, and in “ON” phase, we prospectively assessed clinical data, sleepiness, comorbidities, PD severity (Hoehn&Yahr, HY), motor function (ADLs, UPDRSII and UPDRSIII, Barthel Index, Functional Independence Measure), balance, spirometry, respiratory muscles (MIP/MEP), peak cough expiratory flow (PCEF), continuous night oxygen monitoring, and meters at 6MWT. Of 55 patients analyzed (28 with moderate–severe PD, HY ≥ 2.5), 37% and 23% showed moderate–severe impairment on UPDRSII and UPDRSIII, respectively; 96% had reduced exercise tolerance and severe respiratory muscles impairment (MIP/MEP < 45% pred.); 21.8% showed desaturations during exercise; and 12.7% showed nocturnal desaturations. At multiple regression, low exercise tolerance and low mean nocturnal and exercise-induced saturation correlated with several respiratory and motor function and disability indices (all *p* < 0.03). Exercise tolerance, exercise-induced desaturations, and nocturnal desaturations were extremely frequent in PD patients and were worse in more severe PD patients. This suggests considering a combined role for motor and respiratory rehabilitation in these patients.

## 1. Introduction

Parkinson’s Disease (PD) is one of the most frequent neurodegenerative diseases diagnosed [1], affecting 1–2% of individuals over age 60. However, little is known about the association between PD and ventilatory dysfunction [2]. Restrictive ventilatory defects are commonly found in PD patients at routine spirometry [3,4] but are reversible with levodopa administration [5]. Respiratory muscle strength, airway occlusion pressure at 0.1 s (P0.1) [6], and some lung function parameters are impaired from the early stages of PD onward, with bradykinesia and rigidity being the cardinal signs that correlate most strongly with their impairment [7]. Respiratory muscle weakness may be an important factor in the respiratory complications observed in PD [8,9,10,11]. Respiratory muscle strength training, such as the use of expiratory flow acceleration EFA^®^ technology [12], has the potential to improve expiratory muscle strength, reducing the risk of respiratory complications. 

In PD patients, an increased prevalence of obstructive sleep apnea (OSA) has been demonstrated [13], but the long-term outcomes of disordered breathing events during sleep have not been adequately studied in PD in terms of their real impact on prognosis [14]. Dyspnea is a specific symptom in Parkinson’s diseases [15] experienced by a significant proportion of PD patients and results in limitations of physical ability and a reduced quality of life [7,16,17]. 

To our knowledge, however, no studies have evaluated the role of deranged respiratory components (e.g., oxygen desaturation) during exercise nor at night in patients affected by PD. We hypothesized that PD would be characterized by important exercise intolerance and oxygen desaturation, both during exercise and at night, and that there would be a significant relationship between exercise intolerance, motor impairment, and diurnal and nocturnal respiratory impairment. This could influence the usual rehabilitative treatment in these patients. Thus, we investigated exercise tolerance, oxygenation during exercise as well as nocturnal oxygen saturation in patients with PD.

## 2. Materials and Methods

This was a prospective observational study on patients diagnosed with PD admitted to two different Neuro-Rehabilitation units of the Istituti Clinici Scientifici Maugeri for rehabilitation. The protocol was approved by the Ethical Committee of the ICS Maugeri (2240 CE–9 October 2018). All patients gave their informed consent for the scientific use of their clinical data. 

### 2.1. Patients

**Figure 1:**
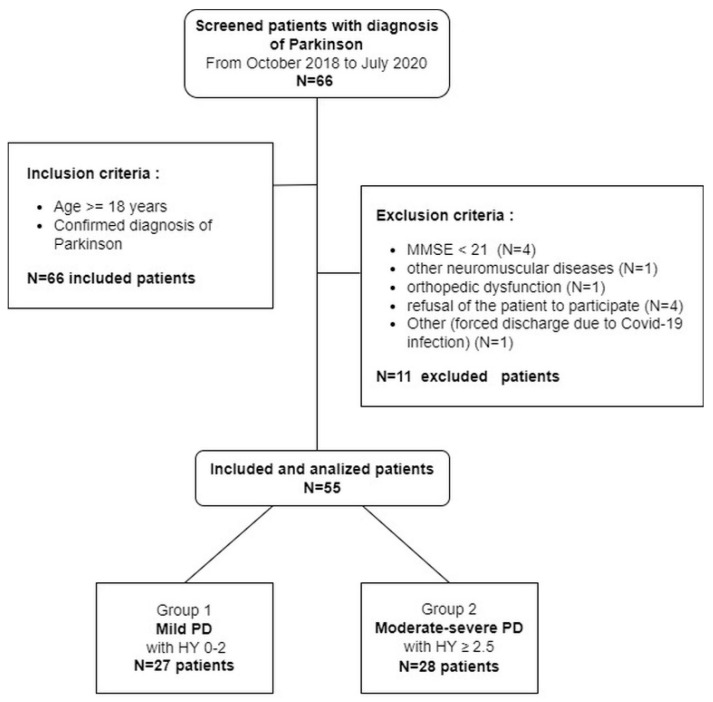
Flowchart of the study. Legend: MMSE = Mini-Mental State Examination; PD = Parkinson’s disease; HY = Hoehn and Yahr staging scale.

Consecutive adult patients (aged > 18 years), of both sexes, with a diagnosis of Parkinson’s disease communicated to the patient at the neurological visit, and who were admitted to the Neuro-Rehabilitation units of ICS Maugeri Lumezzane (Brescia, Italy) and Castel Goffredo (Mantova, Italy) in the period October 2018–July 2020 for a standard inpatient rehabilitation program, were enrolled in the study. Based on patient referrals to the two facilities, we expected to recruit approximately 30 patients/year from each Neuro-Rehabilitation unit. However, due to COVID-19 infection and the consequent hospital reorganization giving priority to patients not attending rehabilitation, to reach the planned number of enrolled PD patients, we extended the original 12-month recruitment period to 20 months. Patients were excluded if they had signs of cognitive impairment, i.e., score < 21 on the Mini-Mental State Examination (MMSE) [18], diaphragm disease, presence of other neuromuscular pathologies, cancer or other terminal diseases, if they were unable to perform a walking test, or refused consent to participate in the study (see study flowchart, Figure 1).

The rehabilitation program was individually tailored to each patient, and possible components of global motor training were stretching exercises; passive, active-assisted, and active mobilization exercises; neuromuscular strengthening exercises; exercises for posture control; walking training with and without external cueing or on a treadmill; lung expansion breathing exercises; exercises for the improvement of language; communication and writing; exercises for dysphagia [19]. 

### 2.2. Measurements

On day 1, the neurologist visited the patient, evaluated the previous prescribed therapy, and, if necessary, varied it based on the symptoms and clinical evaluation. The evaluations performed for this study started 15 days after admission, with the patient in a condition of clinical stability. We assessed patients during the “ON” phase (clinically evaluated and confirmed by the UPDRSIII assessment). The assessment included: anthropometric data (age, sex, weight, body mass index), smoking habits (smoker, non-smoker, ex-smoker), type and severity of comorbidities (CIRS 1 and 2 [20], PD severity (Hoehn and Yahr, HR) [21], motor experiences of daily living (UPDRSII), and PD-related motor examinations (UPDRSIII) [22,23]. Disability on Barthel Index [24] and Functional Independence Measure (FIM) [25], balance on Berg Balance scale [26], and sleepiness on Parkinson’s Disease Sleep Scale 2 (PDSS) [27], and Epworth Sleepiness scale (ESS) [28] were also assessed. Respiratory measurements included spirometry (forced expiratory volume in 1 s (FEV_1_)% pred., forced vital capacity (FVC)% pred., FEV_1_/FVC, peak expiratory flow (PEF) [29] (Spirodoc, MIR Medical International Research, New Berlin, WI, USA), respiratory muscle function (maximal inspiratory pressure (MIP% pred.) and maximal expiratory pressure (MEP% pred.)) [30] (MicroRPM, CareFusion, Hoechberg, Germany), peak cough expiratory flow (PCEF) [31] (Spirodoc, MIR Medical International Research, USA with Ambu Ultraseal mask), continuous night oxygen monitoring (mean SpO_2_%, oxygen desaturation index (ODI), desaturation time (T90%), and the number of patients with nocturnal desaturations identified by T90 > 30%) (Spirodoc, MIR Medical International Research, New Berlin, WI, USA). The following day (day 16), patients performed the 6 min walk test (6MWT) [32] with the distance walked measured in meters and % of predicted meters, oximetry at rest (SpO_2_%), mean SpO_2_%, and minimum SpO_2_ (nadir) (Spirodoc, MIR Medical International Research, USA). The numbers of patients with reduced (<80% of predicted meters) and severely reduced (<50% of predicted meters) exercise tolerance and the number of patients with oxygen desaturation identified by a delta saturation (basal–mean)% > 4% [33] were recorded. 

### 2.3. Statistical Analysis

As this was an observational cohort study, we did not calculate the representative sample size. Data were evaluated for Gaussian distribution (Shapiro–Wilk test) and then summarized as mean ± standard deviation (SD), or median, and 1st–3rd quartile (for quantitative variables) and frequencies (for categorical variables) before applying statistical analysis using the Graph Pad Software (Prism 4, San Diego, CA, USA) and the R programming language (Vienna, Austria, 2018) [34]. 

Depending on PD severity assessed by the HY, patients were divided into two groups based on symptoms and impairment of balance: group 1 (mild PD) with unilateral symptoms and no impairment of balance (HY 0–2) and group 2 (moderate–severe PD) with moderate-to-severe bilateral symptoms and balance or gait disabilities (HY ≥ 2.5) [35]. The non-parametric (Wilcoxon) test and Pearson chi-squared test (applying the Monte Carlo correction in the case of low numbers) were applied for data comparison between the two groups. The risk of association of certain variables to a more severe PD stage was evaluated by the odds ratio (OR) in regard to all appropriate baseline variables. 

The correlations between demographic, anthropometric, clinical, and respiratory variables versus motor ADLs (UPDRS II), motor function (UPDRS III), and exercise tolerance (6MWT, expressed as % of predicted) were analyzed by Spearman correlation (Table S1 Supplementary Material). Multiple regression analysis was applied considering three different dependent variables: 6MWT (% of predicted), night mean SpO_2_, and 6MWT mean SpO_2_. A selection of clinical predictors evaluated by Spearman’s correlation (see Supplementary Table S1) was used as covariates. For all analyses, a value of *p* < 0.05 was considered as statistically significant.

## 3. Results

Fifty-five patients were analyzed, and their clinical characteristics (of the overall sample and of the two subgroups based on PD severity) are presented in Table 1. Patients were elderly, with an even male/female distribution, and of normal weight. The mean disease duration was 7.8 ± 4.7 years, and 96% of patients showed reduced exercise tolerance at the 6MWT test with a median performance (overall sample) of 365 m (260–465), equal to 56% pred. (39–65). Concerning prescribed medications, 90% of patients were on levodopa, 45% on dopamine agonists, and 38% on levodopa plus dopamine agonists. The average levodopa dosage/day was 550 mg. These patients had severe impairment in respiratory muscles (MIP and MEP <45% pred.); 12.7% had nocturnal desaturations and 21.8% had desaturation during exercise with a medium level of dyspnea (>2) at the end of the exercise. According to the UPRDS triangulation cut-off values reported by Martinez-Martin [36], 37% and 23% presented moderate-to-severe impairment on UPDRSII and UPDRSIII, respectively. Symptoms related to sleepiness (PDSS and Epworth) suggested a moderate level of impairment.

The group with moderate–severe PD (28 patients with HY ≥ 2.5) had more comorbidities and a worse level of ADLs, motor function and disability, balance, FVC% pred., PCEF, nocturnal oxygen saturation, exercise tolerance, and PDSS (Table 1).

Of note, from OR analysis, (1) the risk of having FVC% pred. ≤80% was about 7 times higher (OR 6.944; *p* < 0.02; 95% CI = 1.354–35.607) in the group with moderate–severe PD compared to mild PD; (2) the risk of having severely reduced exercise tolerance (defined as 6MWT% pred. < 50%) was about 19 times higher (OR 19.1667; *p* < 0.0001; 95% CI = 4.7269–77.7163) in moderate–severe PD than in mild PD.

**Figure 2:**
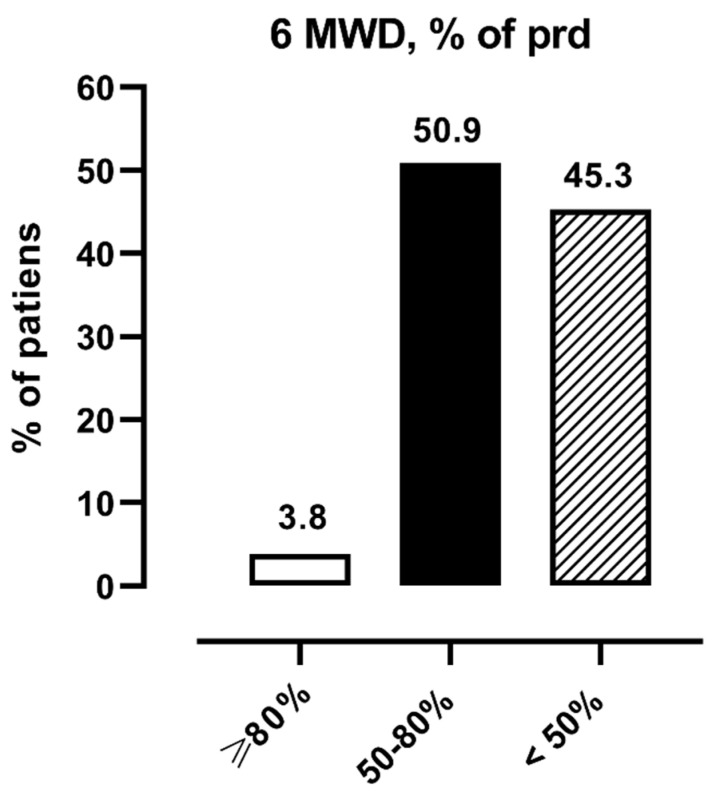
Distribution of patients according to level of performance at 6MWT: normal (≥80% pred.), moderately reduced (50–80% pred.) and severely reduced (<50% pred.). Legend: 6MWT = 6 min walking distance.

Figure 2 shows the distribution of patients according to level of performance at 6MWT: normal (≥80% pred.), moderately reduced (50–80% pred.), and severely reduced (<50% pred.).

At multiple regression, high disability (Barthel Index) and a low PCEF were significant predictors (both *p* < 0.05) of low exercise tolerance evaluated by 6MWT (Table 2, R^2^ = 0.465, *p* < 0.0001) in PD patients. The correlation of lower CIRS1 and higher FVC% pred. with exercise tolerance was close to statistically significant. High BMI, presence and severity of comorbidities, and low mean saturation during exercise were significant predictors (all *p* < 0.006) of low mean nocturnal saturation (Table 2, R^2^ = 0.515, *p* < 0.0001), while female sex, high disability, low age, BMI, FEV_1_% pred., and mean nocturnal saturation were significant predictors (all *p* < 0.04) of low mean saturation during exercise (Table 2, R^2^ = 0.430, *p* < 0.0001).

**Figure 3:**
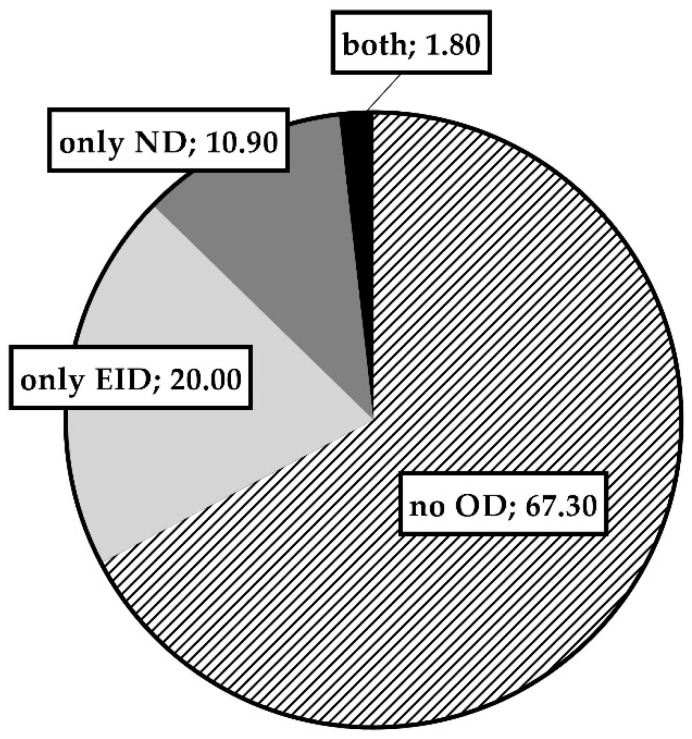
Percentage of patients not presenting oxygen desaturation (no OD), presenting exercise-induced desaturation only (only EID), nocturnal desaturation only (only ND), and both exercise and nocturnal desaturation (both).

Figure 3 shows the percentage of patients not presenting oxygen desaturation (67.3%), those with exercise-induced desaturation only (20.0%), and those with nocturnal desaturation only (10.9%). One patient presented both exercise and nocturnal desaturation (1.8%).

CIRS 1 severity was different among groups (ANOVA, *p* = 0.0044). Patients with nocturnal desaturation had a significantly higher number and severity of comorbidities (CIRS1) compared to patients without desaturation (*p* = 0.0084). A significant difference was also found for level of FVC% pred. (ANOVA, *p* = 0.027) but without significant differences in the post hoc analysis.

### Correlations

Further correlations between ADL level, motor function, and exercise tolerance with different clinical functional scales are reported in Table S1 Supplementary Materials. As expected, a strong and positive correlation emerged for all three variables mentioned above versus disability measured by Barthel Index, FIM, and Balance Berg scale. Patients with worse ADL level and motor function had a worse expiratory flow, cough ability, number of nocturnal desaturations, nocturnal time spent with <90% of saturation, and diurnal somnolence. Patients with lower exercise tolerance had lower FEV_1_% pred. and FVC% pred. at spirometry, lower cough ability, and more diurnal somnolence measured with PDSS.

## 4. Discussion

Our study has shown that 96% of PD patients have exercise intolerance (severe in 45%), 21.8% have exercise-induced oxygen desaturation, 12.7% have nocturnal desaturation, and the severer the PD is, the more compromised these parameters are. The risk of low forced expiratory volumes and reduced exercise tolerance was particularly high in patients with more severe PD. Low exercise tolerance was related to high disability and low cough peak; low mean nocturnal saturation was related to high BMI, a higher number of comorbidities and low mean oxygen saturation during exercise; low mean exercise saturation was related to female sex, high disability, low age, BMI, and forced respiratory volumes. 

PD is characterized by motor symptoms, rigidity and tremor [37], and fatigue. Problems with gait start with shuffling and short steps, difficulty turning around whilst standing, postural instability, and risk of falling. The reduced physical capacity is a combination of muscle strength, muscle tone, muscle endurance, exercise tolerance and joint mobility [38], poor timing of velocity, and reduced muscle power [39]. Although these symptoms are well known, they represent a huge burden for PD patients, compromising their daily activities [40] and quality of life [41,42]. PD patients experience problems in multiple domains (function, ability, activities, ADLs, social participation, and sleep) that can either be a consequence of the disease itself, or an effect of the PD medication, timing of drug administration, and inactivity [43]. 

Studies have examined aerobic capacity in subjects with PD [44,45,46]. Interestingly, Canning [47] reported no correlation between disease severity and VO_2_peak in individuals with mild to moderate PD while VO_2_peak values were found generally 20% lower than age-matched controls without PD studied [48].

In a recent review [46] on the most suitable clinical exercise testing in PD, the authors cited the 6MWT test as an important field test. The 6MWT test was used as an outcome measure after treadmill training [49] or limb strength training, as well as aerobic training and incremental aerobic cycling [45]. In both papers, distance walked was lower than predicted before, but improved after, rehabilitation.

Surprisingly, no studies have analyzed the relationship between 6MWT and PD severity or oxygen desaturation as a possible limiting factor during the 6MWT test; these questions were among the aims of our study. Our data confirm an important reduction in exercise tolerance (56% of predicted meters), with a further drop (41% of predicted meters) in the more compromised PD stages, and that about 21.8% of patients presented oxygen desaturation during exercise. Our data also show that more than 45% of patients had a severely compromised exercise tolerance. It is not surprising that high general disability and number of comorbidities (close to limit of significance) were correlated with reduced exercise tolerance. 

Respiratory dysfunction remains one of the most common causes of death in patients with complicated PD. Pulmonary restrictive defects are common in PD patients and are evident at routine spirometry monitoring [5] both in “on” and “off” states of disease [4], though they are reversible with levodopa administration [4,5]. In addition, our data have confirmed a pathological restrictive pattern in more severe PD patients. The restrictive dysfunction seems not related to tremor, rigidity, or bradykinesia but to the rigidity and musculoskeletal limitations of the vertebral column probably induced by a chronic anomalous posture [50]. Of note, the derangement in forced volumes may have contributed to the reduction of exercise tolerance in our patients. Respiratory muscle weakness may be an important factor in the respiratory complications in PD [6,9] as confirmed by our data, which showed a strong reduction of maximal inspiratory (43% of predicted value) and expiratory pressures (40% of predicted value) independently of the level of disease.

Maximal expiratory flow after expiration may be significantly below normal [50] in PD patients; our results confirm an important reduction in cough ability, which was particularly pronounced in the more severe patients. As for FVC% pred., the derangement in cough ability was found to be correlated to the reduction of exercise tolerance.

Sleep alterations in PD may occur even in the early stages [14,51,52], while sleep microarchitecture improves with levodopa [51]. The prevalence of sleep-disordered breathing seems greater in PD patients compared to age- and gender-matched controls [13]. Some studies have shown that disordered breathing events in sleep are associated with less significant oxyhemoglobin desaturation in PD [14]. In our study, 12.7% of patients presented nocturnal desaturation, while in more severe ones the rate was 17.6% with worse symptoms of sleepiness. The desaturation index, i.e., time spent below 90% of saturation, and mean saturation were clearly more compromised in the more severe PD patients. It is not surprising that low mean nocturnal saturation was related to high BMI and comorbidities, but somewhat less expected was our finding that low mean nocturnal saturation was related to low mean exercise-induced saturation. Of note, only one patient presented both exercise and nocturnal desaturation; there were more patients with exercise desaturation only (20.0%) than with nocturnal desaturation only (10.9%).

### 4.1. Limitations and Strengths

Due to gait problems, increased risk of falling, and variable response to dopaminergic medications, the patients may have performed below their real level of exercise. Those with more severe PD would also likely have had less reproducible measurements during the 6MWT test based on wider motor fluctuations. 

One strength of the study is that it is the first to have investigated, in a single study, exercise tolerance and oxygen saturation during exercise as well as at night. Another strength of the study is that n = 55 could be considered a relatively good number of patients with a high number of neurological and respiratory measurements at the same time. All participants, with different disease severity, were well characterized, and all were receiving optimal medical management, especially regarding postural stability [53].

### 4.2. Clinical Implications

Healthcare personnel should pay careful attention to the threefold relationship between exercise tolerance and nocturnal and exercise desaturation, and consider the common opportunity of motor (activation, deconditioning, straight, training) and respiratory rehabilitation programs (oxygenation, nocturnal CPAP, cough assisted devices). The complexity of PD disability will be cared for a multidisciplinary manner obtaining synergic and multiplicative effects. Simple evaluations designed to asses respiratory muscle function, exercise desaturation, and nocturnal desaturation can permit an individualized approach in progressively neurological diseases such as PD.

## 5. Conclusions

In PD patients, exercise tolerance, exercise-induced desaturations, and nocturnal desaturations are below normal in 96%, 21.8%, and 12.7% of patients, respectively, and the severer the disease, the more compromised the values are. Hence, not only do comorbidities, age, nutritional status, high disability, and reduced respiratory function have an influence on exercise tolerance, but exercise-induced and nocturnal mean oxygen saturation seem to play a role as well. Nocturnal and exercise-induced mean saturation levels would appear to be interrelated. Therefore, exercise and physical activity prescription in PD needs special attention regarding the oxygen desaturation component. This suggests considering a combined role for motor and respiratory rehabilitation in these patients. 

## Figures and Tables

**Table 1:** Characteristics of PD patients at admission: results for the overall sample and mild vs. moderate–severe subgroups.

	Overall n = 55	Group 1: HY 0–2 n = 27	Group 2: HY ≥ 2.5 n = 28	*p*	Delta	95% CI
Age, years	73 (68–79)	72 (67–76)	75 (70–79)	0.1878		
Male, %	53	59	46	0.4948		
BMI, Kg/m^2^	25.2 (22.9–29.9)	24.3 (22.8–27.1)	26.7 (23.4–31.3)	0.0875		
Smokers, yes%	3.7	3.7	3.7	1.0000		
Ex-smokers, yes%	18.9	19.2	18.5	1.0000		
CIRS1, score	1.5 (1.3–1.8)	1.4 (1.2–1.5)	1.6 (1.4–1.9)	**0.0022**	0.3	0.1; 0.4
CIRS2, score	2.0 (2.0–4.0)	2.0 (1.0–3.0)	3.0 (2.0–4.2)	**0.0005**	1.0	1.0; 2.0
Comorbidities, yes%	64.2	65.4	63.0	1.0000		
COPD, yes%	7.5	3.8	11.1	0.6105		
CHF, yes%	1.9	3.8	0	0.4906		
Diabetes, yes%	9.4	3.8	14.8	0.3507		
Hypertension, yes%	34.0	26.9	40.7	0.3869		
Others, yes%	43.4	34.6	51.8	0.2709		
MMSE, score	27.4 (25.3–3.0)	28.0 (25.3–30.0)	27.0 (25.6–28.0)	0.2263		
UPDRSII, score	9 (6–18)	7 (3–9)	15 (9–22)	**0.0004**	7	3; 12
UPDRSIII, score	17 (11–28)	13 (10–17)	26 (16–44)	**0.0006**	14	5; 25
Barthel Index, score	95 (80–100)	100 (97–100)	80 (60–95)	**<0.0001**	−20	−25; −10
FIM, score	113 (90–126)	126 (104–126)	91 (84–121)	**0.0006**	−18	−32; −6
Berg balance, score	44 (35–50)	47 (42–50)	38 (33–47)	**0.0218**	−6	−12; −1
FEV_1_, % predicted	95 (84–109)	103 (90–113)	93 (77–106)	0.1947		
FVC, % predicted	94 (81–109)	98 (88–111)	91 (77–104)	**0.0338**	−12	−24; −1
FEV_1_/FVC, %	79 (75–83)	77 (74–81)	81 (76–86)	0.1424		
PEF, % predicted	84 (65–104)	93 (69–108)	78 (63–98)	0.1572		
MIP, % predicted	43 (34–51)	45 (37–51)	41 (33–50)	0.3773		
MEP, % predicted	40 (30–50)	42 (29–56)	36 (31–48)	0.5275		
PCEF, L/min	300 (218–405)	366 (247–448)	282 (208–308)	**0.0168**	−95	−148; −18
Night SatO_2_ ODI, n/h	8.3 (2.9–15.3)	4.3 (0.8–10.2)	13.5 (6.6–22.8)	**0.0032**	7.3	2.8; 12.7
Night SatO_2_ T90, %	1.40 (0.15–8.30)	0.40 (0.05–1.19)	3.95 (1.78–16.50)	**0.0002**	2.60	1.34; 9.30
Night SatO_2_ mean, %	93.8 (92.3–95.5)	94.9 (93.7–96.1)	92.6 (91.9–94.6)	**0.0016**	−2.1	−3.3; −0.8
Night SatO_2_ nadir, %	83.0 (75.5–86.2)	84.0 (76.0–88.0)	82.0 (75.0–85.0)	0.3684		
Nighttime Desaturators, n (%)	7 (12.7)	2 (7.4)	5 (17.6)	0.4216		
6MWT, % predicted	56 (39–65)	63 (57–68)	41 (35–49)	**<0.0001**	−20	−26; −12
6MWT SatO_2_ bas, %	96.0 (94.7–97.0)	96.5 (95.4–97.1)	95.7 (93.8–96.5)	**0.0410**	−1.0	−2.0, 0.0
6MWT SatO_2_ mean, %	94.5 (91.4–95.59)	94.7 (92.5–96.3)	94.0 (91.0–95.0)	0.2894		
6MWT SatO_2_ nadir, %	91 (86–93)	92 (87–94)	91 (85–93)	0.3219		
6MWT Borg pre, score	0 (0–0)	0 (0–0)	0 (0–0)	0.0950		
6MWT Borg post, score	3 (2–3)	3 (2–3)	3 (2–4)	0.3261		
Exercise Desaturators, n (%)	12 (21.8)	7 (25.9)	5 (17.9)	1.0000		
PDSS, score	102 (85–123)	110 (95–126)	92 (79–115)	0.1112		
Epworth, score	5 (3–9)	5 (3–8)	5 (3–12)	0.3804		

Legend: values expressed as median (interquartile range) or percentage. HY = Hoehn and Yahr staging scale; BMI = Body mass index; COPD = Chronic obstructive pulmonary disease; CHF = Chronic heart failure; CIRS = Cumulative Illness Rating Scale severity (CIRS1) and comorbidity (CIRS2); MMSE = Mini-Mental State Examination; UPDRS = Unified Parkinson’s Disease Rating Scale; FIM = Functional Independence Measure; FEV_1_ = Forced expiratory volume at 1 s; FVC= Forced vital capacity; PEF = Peak expiratory flow; MIP = Maximal inspiratory pressure; MEP = Maximal expiratory pressure; PCEF = Peak cough expiratory flow; ODI = Oxygen desaturation index; 6MWT = 6 min walking distance; 6MWT = 6-min walk test; PDSS = Parkinson’s Disease Sleep Scale. In bold font, *p* < 0.05.

**Table 2:** Multiple regression analysis on outcomes as dependent variables: exercise tolerance (expressed as 6MWT% of predicted value), Night mean SpO_2_, and 6MWT mean SpO_2_.

Dependent Variables	Independent Variables	Estimated Beta	Standardized Beta	Adjusted R^2^	*p*-Value
6MWT% of pred.				**0.465**	<0.0001 ***
	(Intercept)	25.948			0.1904
	CIRS1	−11.918	−0.258		0.0560 °
	Barthel Index	0.244	0.294		0.0246 *
	FVC% pred.	0.159	0.262		0.0683 °
	PCEF (L/min)	0.029	0.229		0.0450 *
Night mean SpO_2_				**0.515**	<0.0001 ***
	(Intercept)	80.101			<0.0001 ***
	BMI	−0.138	−0.290		0.0056 **
	CIRS1	−3.2541	−0.438		0.0002 ***
	6MWT mean SpO_2_	0.240	0.326		0.0031 **
6MWT mean SpO_2_				**0.430**	<0.0001 ***
	(Intercept)	9.734			0.5930
	Sex M	1.791	------		0.0228 *
	Age	0.109	0.236		0.0431 *
	BMI	0.190	0.292		0.0150 *
	Barthel Index	0.049	0.268		0.0265 *
	FEV_1_% pred.	0.065	0.363		0.0023 **
	Night mean SpO_2_	0.651	0.479		0.0006 ***

Legend: 6MWT = 6 min walking distance; CIRS1 = Cumulative Illness Rating Scale severity; FVC = Forced vital capacity; PCEF = peak cough expiratory flow; BMI = Body mass index 6MWT = 6-min walk test; FEV_1_ = Forced expiratory volume at 1 s; *** = <0.001; ** = <0.01; * = <0.05; ° = <0.1. Gaussian distribution of residuals was evaluated. In bold font, *p* and R^2^ of the different models.

## Data Availability

Anonymized data materials will be made publicly available at https://www.zenodo.org (accessed on 16 November 2021).

## Supplementary Materials

The following are available online at https://www.mdpi.com/article/10.3390/ijerph182312298/s1, Table S1: Spearman correlations of Motor ADL, Motor function, and Exercise tolerance with all other variables.
