# Supplementary material for: Exercise Intolerance and Oxygen Desaturation in Patients with Parkinson’s Disease: Triggers for Respiratory Rehabilitation?
Source: Int J Environ Res Public Health. 2021 Nov 23;18(23):12298. doi: 10.3390/ijerph182312298 (PMC8656612; doi:10.3390/ijerph182312298)
Supplement: Supplementary file 1 [file ijerph-18-12298-s001.zip › ijerph-1448608-supplementary.pdf]

SUPPLEMENTARY MATERIALS

Table S1. Spearman correlations of Motor ADL, Motor function and Exercise tolerance with all other variables

	**Motor ADL (UPDRS II)**		**Motor function (UPDRS III)**		**Exercise (6MWT_meters %prd)**	
	**Spearman r**	**P (two-tailed)**	**Spearman r**	**P (two-tailed)**	**Spearman r**	**P (two-tailed)**
**BMI (kg/m^2)**	0.1597	0.2441	0.1189	0.3873	--------	--------
**Age, years**	0.1998	0.1435	0.1597	0.2441	--------	--------
**CIRS1, score**	**0.6255**	**<0.0001**	**0.5511**	**<0.0001**	**-0.5740**	**<0.0001**
**CIRS2, score**	**0.5124**	**<0.0001**	**0.4828**	**0.0002**	**-0.5947**	**<0.0001**
**UPDRS II, score**	--------	--------	--------	--------	**-0.5336**	**<0.0001**
**UPDRS III, score**	--------	--------	--------	--------	**-0.4734**	**0.0003**
**Barthel Index, score**	**-0.7088**	**<0.0001**	**-0.6018**	**<0.0001**	**0.6574**	**<0.0001**
**FIM, score**	**-0.7067**	**<0.0001**	**-0.6426**	**<0.0001**	**0.5250**	**<0.0001**
**Berg balance, score**	**-0.3959**	**0.0028**	**-0.3359**	**0.0122**	**0.4203**	**0.0017**

**FEV1 % pred.**	-0.1520	0.2679	-0.1969	0.1495	**0.3778**	**0.0053**
**FVC % pred.**	-0.1040	0.4497	-0.1302	0.3433	**0.4449**	**0.0008**
**FEV1/FVC**	-0.1382	0.3144	-0.2502	0.0654	-0.0716	0.6101
**PEF % pred.**	**-0.4613**	**0.0004**	**-0.4090**	**0.0019**	**0.4266**	**0.0014**
**MIP, % pred.**	**-0.5834**	**<0.0001**	**-0.4290**	**0.0012**	**0.2860**	**0.0398**
**MEP, % pred.**	-0,2211	0.1048	-0.1935	0.1569	**0.2756**	**0.0458**
**PCEF (L/min)**	**-0.4870**	**0.0002**	**-0.3638**	**0.0063**	**0.4818**	**0.0002**
**ODI, n**	**0.3056**	**0.0246**	**0.3322**	**0.0141**	**-0.3176**	**0.0217**
**Nocturnal $SatO_2$ T90, %**	**0.4133**	**0.0017**	**0.4443**	**0.0007**	**-0.4441**	**0.0009**
**Nocturnal $SatO_2$ mean, %**	**-0.4770**	**0.0002**	**-0.4185**	**0.0015**	**0.4236**	**0.0016**
**Nocturnal $SatO_2$ nadir, %**	-0.2567	0.0662	**-0.3530**	**0.0102**	0.2534	0.0758
**6MWD $SatO_2$ bas, %**	**-0.3881**	**0.0037**	**-0.2988**	**0.0282**	0.2544	0.0660
**6MWD $SatO_2$ mean, %**	**-0.2976**	**0.0305**	**-0.3403**	**0.0127**	0.2166	0.1193
**6MWD $SatO_2$ nadir, %**	-0.2335	0.0924	**-0.3216**	**0.0189**	0.2117	0.1280
**PDSS**	-0.1661	0.2441	-0.1364	0.3400	**0.3170**	**0.0265**
**Epworth**	**0.3485**	**0.0105**	**0.3037**	**0.0270**	-0.0567	0.6926

Legend: BMI=Body mass index; CIRS=Cumulative Illness Rating Scale severity; UPDRS= Unified Parkinson's Disease Rating Scale; FIM= Functional Independence Measure; FEV1=Forced expiratory volume at 1 second ; % prd=% of predicted value; FVC= Forced vital capacity; PEF=peak expiratory flow; MIP=maximal inspiratory pressure; MEP=maximal expiratory pressure; PCEF=peak chough expiratory flow; ODI=apnea-hypopnea index; 6MWD=6-min walking distance; PDSS=Parkinson's Disease Sleep Scale 2.
